# Genetic and Environmental Contributors To Congenital Heart Disease

**DOI:** 10.1007/s11936-025-01091-5

**Published:** 2025-05-26

**Authors:** Talita Z. Choudhury, Benjamin L. Gilbert, Vidu Garg

**Affiliations:** 1 Center for Cardiovascular Research, The Heart Center, Abigail Wexner Research Institute, Nationwide Children’s Hospital, Room WB4275, Columbus, OH 43205 USA; 2 Department of Molecular Genetics, The Ohio State University, Columbus, OH USA; 3 Department of Pediatrics, The Ohio State University, Columbus, OH USA

**Keywords:** Congenital heart disease, Cardiovascular genetics, Gene-environment interaction, Genetic etiology, Environmental etiology, Gene therapy

## Abstract

**Purpose of Review:**

Paradigms surrounding congenital heart disease (CHD) etiology represent an evolving area of study. Traditionally, genetic causes of CHD have been classified into chromosomal abnormalities, copy number variation, and single-gene disorders, while environmental contributors include external and intrinsic maternal factors that impair cardiac development. Here, we summarize established causes of CHD and highlight emerging insights into CHD pathogenesis that may inform future treatment options.

**Recent Findings:**

Recent advancements in next-generation sequencing technologies have uncovered novel genetic etiologies underlying CHD including oligogenic inheritance and pathogenic noncoding variation. In addition, industrialization and transformation of society has introduced new environmental risk factors that may contribute to CHD. Further, mechanistic insight into both genetic and environmental factors underlying CHD has led to discovery of novel therapeutic strategies.

**Summary:**

New methodologies have greatly improved our comprehension of the heterogeneous mechanisms underlying CHD, catalyzing the discovery of effective therapeutic strategies to reduce CHD incidence.

## Opinion Statement

In 2025, only about a third of the estimated human congenital heart disease (CHD)-causing genes have been validated while most instances of CHD, even heritable cases, have unknown etiologies. Several explanations for this include the influence of the environment, incomplete disease penetrance and the extensive genetic variation within the population. Although a tenth of CHD cases are due to exposure to environmental risk factors, it is likely that most idiopathic CHD is genetic in nature and may include variation in non-coding regulatory elements, oligogenic inheritance and complex gene-environment interaction among other non-Mendelian factors. As next-generation sequencing (NGS) technologies are becoming increasingly advanced, investigating these complex molecular mechanisms underlying CHD will become more accessible. In conjunction with genome-wide association studies (GWAS) in large CHD cohorts, the identification of novel genetic etiologies and mechanisms for CHD will be dramatically accelerated and set the foundation for a bench to bedside approach in the treatment and management of CHD.

## Introduction

Congenital heart disease (CHD) is one of the most prevalent pediatric health challenges, affecting at least 40,000 infants in the United States each year [1, 2]. Advancements in postnatal surgical intervention and palliative care has led to improved survival rates of CHD patients, reflected by the increasing number of adults with CHD. However, the heterogeneity of CHD etiologies and a limited understanding of molecular mechanisms underlying the wide spectrum of CHD phenotypes has precluded efforts to develop effective preventative strategies to decrease the incidence at birth or reduce disease severity [3, 4].

**Fig. 1:**
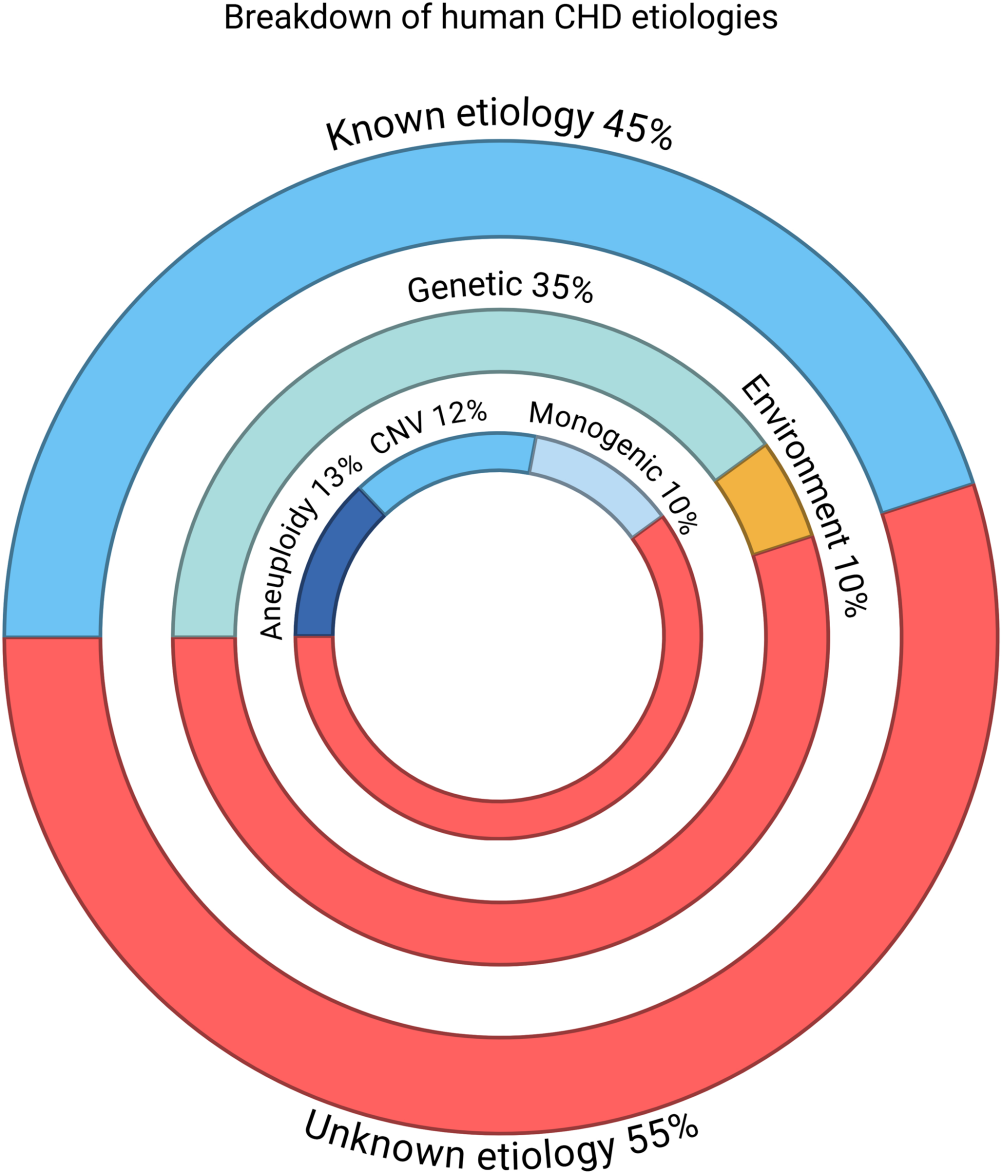
Pie-chart representing the percentage of known and unknown etiologies in CHD cases. Shown from outside in is the proportion of known vs. unknown etiologies, the proportion of genetic vs. environmental etiologies, and the proportion of known genetic etiologies. CNV, copy number variation. All percentages are approximations. Figure made with Biorender

Approximately 45% of CHD cases can be attributed to a known etiologic factor, wherein ~ 35% are due to presence of pathogenic genetic loci and ~ 10% due to exposure to modifiable environmental contributors (Fig. 1) [5–8]. Among the genetic contributors of CHD, chromosomal aneuploidies make up ~ 13% of CHD cases, copy number variation ~ 12%, and monogenic causes of CHD are identified in ~ 10% of CHD cases [9]. Conversely, environmental etiologies that contribute to CHD are also extremely heterogeneous and can be broadly classified into extrinsic and intrinsic factors. Extrinsic environmental factors include maternal exposure to teratogenic agents such as heavy metals, drugs, and nutritional deficiencies. Extrinsic factors are generally considered more modifiable, and the consensus has been to identify and prevent such exposures in order to decrease the associated incidence of CHD. However, intrinsic factors are far more difficult to control, as they relate to maternal physiological aspects such as age, illnesses and infections that affect the intrauterine environment and thereby disrupt cardiac development. Worse still, complex molecular interactions between genetic and environmental factors have long been hypothesized to drive at least a subset of non-Mendelian CHD cases, further compounding our understanding of CHD pathogenesis. Nevertheless, the last three decades have witnessed remarkable progress in the field. Large datasets obtained from multiomics-based investigations using diverse CHD cohorts has led to paradigm shifting views on the genetic architecture underlying the etiology of CHD while novel mechanisms regarding environmental effects on cardiac development are paving the way for therapeutic strategies that may be implemented during pregnancy. In this review, we briefly summarize known genetic and environmental etiologies of CHD, highlight new findings on the etiologic mechanisms underlying CHD, and discuss how these novel insights may translate into effective treatment options to reduce the prevalence of CHD-affected births.

## Genetic Etiologies of CHD

**Fig. 2:**
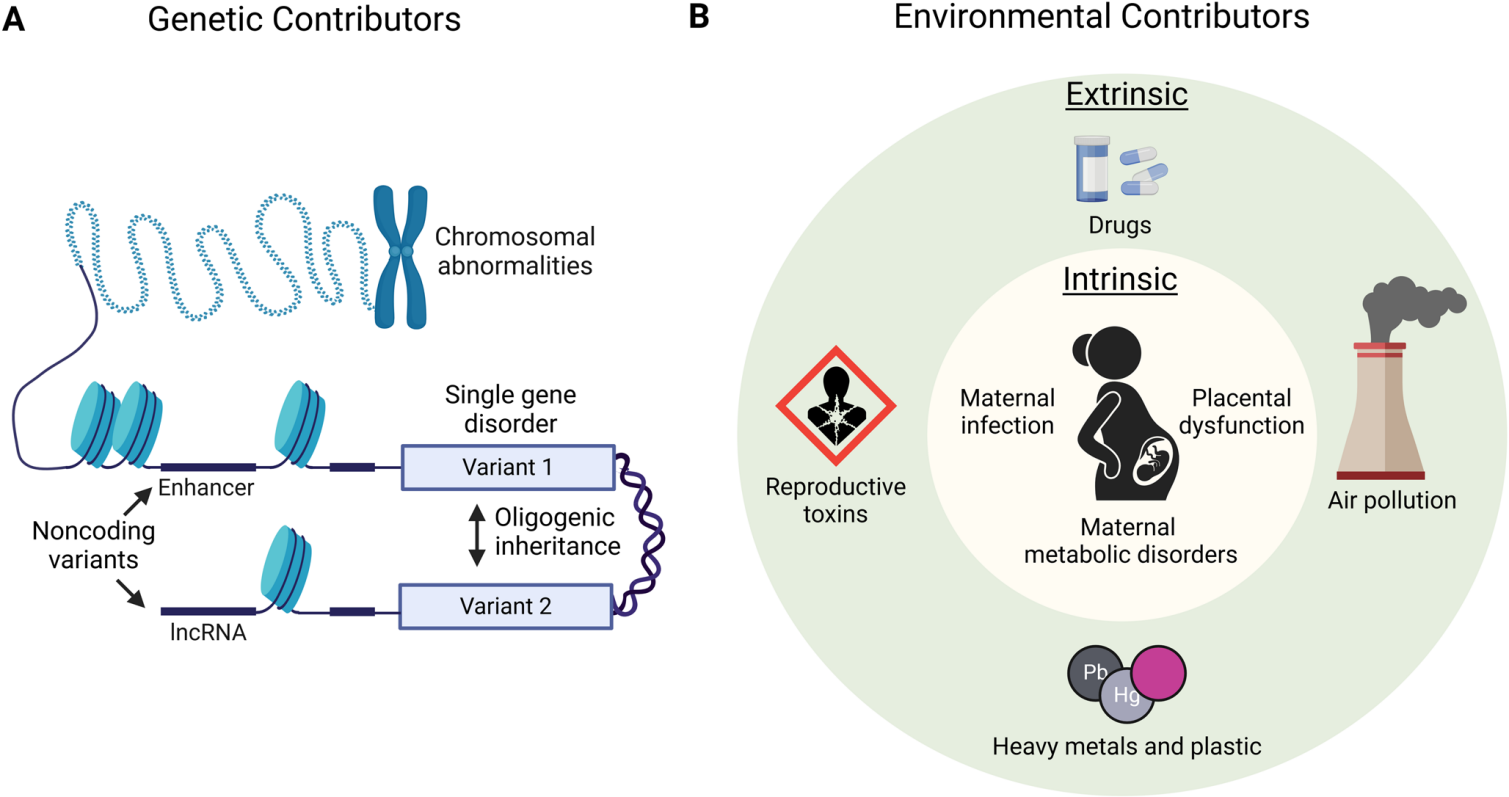
**A** Schematic of genetic etiologies of CHD. **B** Schematic of environmental etiologies of CHD. Figure made with Biorender

The elevated recurrence rate of CHD in affected families highlights the need to identify its underlying genetic factors. In addition to chromosomal abnormalities and copy number variation, several genetic variants linked to CHD were historically thought to follow a monogenic disease inheritance pattern. However, recent technological advances have allowed the identification of novel CHD variants and non-Mendelian genetic mechanisms underlying CHD. In this section, we summarize the established genetic contributors and discuss novel genetic mechanisms underlying CHD (Fig. 2A).

### Established Genetic Contributors of CHD

Chromosomal abnormalities and copy number variation (CNV) have been strongly associated with syndromic CHD, owing to disrupted expression and dosage of crucial developmental genes located within the affected loci. Chromosomal aneuploidies and larger CNVs can be identified using karyotyping and chromosomal microarray, allowing clinicians adequate time to devise plans for postnatal treatments and care. On the other hand, the high recurrence rate of CHD in family members have led to the identification of several single gene disorders that contribute to both syndromic and isolated CHD. Historically identified through linkage analysis, the recent application of next generation sequencing (NGS) technologies has dramatically accelerated the identification of novel genetic variants that may contribute to CHD. Among the most notable advancements is the establishment of the Pediatric Cardiac Genomics Consortium (PCGC), a multi-institutional initiative funded by the NIH/NHLBI, aimed at leveraging NGS to uncover novel genetic etiologies of CHD [10, 11]. Following suit, various other pediatric health institutions have also begun implementation of NGS approaches to aid identification of causal CHD variants [12–15]. As such, rigorously curated online resources are available that display known CHD genes including the Victor Chang CHDgene database and the Online Mendelian Inheritance of Man (OMIM) database [16, 17]. Currently, CHDgene reports 142 high confidence protein coding CHD genes (variants/defects identified in multiple patients); about a third of the minimum estimated number of total human CHD genes [6]. Despite this, known genetic contributors are identified in less than half of all CHD cases, underscoring the need to discover novel etiologic disease mechanisms.

## Novel Genetic Mechanisms Underlying CHD

Discovery of novel mechanisms that contribute to CHD requires an acute knowledge of the biology of cardiac development and disease, driving researchers to investigate the molecular regulation and mechanisms of CHD genes in a developmental and gene regulatory context. When combined with large datasets generated from multiomics-based studies using diverse CHD patient cohorts and animal models of cardiac development, several investigations have led to identification of non-Mendelian factors that contribute to CHD. This includes oligogenic inheritance patterns and variation in non-coding regulatory regions, amongst likely many others. Below we highlight key findings in this area.

### Biallelic and Oligogenic Inheritance

Traditionally, most genetic CHDs exhibit an autosomal dominant inheritance pattern, resulting from haploinsufficiency due to loss-of-function variation in CHD genes. However, complementary inheritance of rare homozygous variants have also been identified to contribute to CHD. This includes a recent report of biallelic inheritance of two recessive phospholipase D1 (PLD1) loss-of-function variants leading to right-sided valvular disease and cardiomyopathy [18]. Interpretation of such rare, recessive variants is challenging as disease manifestation depends on co-inheritance of two complementary variants and thus, can remain undetected in CHD cohorts when present in a heterozygous state. Alternatively, the presence of protective variants can also mask the effect of deleterious variants as shown by Teekakirikul et al. [19]. In this study, a potentially deleterious *TPM1* variant was identified in a family with atrial septal defects (ASD). Although this variant was found to be embryonic lethal in mice, patient induced pluripotent stem cell (iPSC) derived cardiomyocytes harboring this genetic variant showed very mild defects, suggesting disease suppression. In fact, a common *TLN2* variant that was coinherited with the *TPM1* variant was able to rescue embryogenesis in mice but resulted in ASD, recapitulating the human phenotype, suggesting a protective interaction with TPM1. Interestingly, a recent study by Ramirez et al. using in vivo models identified evidence of a buffering mechanism that exists during the development of the aortic arch [20]. Genotypes that impair the development of the second heart field (SHF)-derived pharyngeal arch artery (PAA) endothelium through conditional deletion of an essential endothelial gene, *VEGFR2*, still maintain a VEGFR2 + PAA endothelium. It was revealed that this PAA endothelium was derived from venous endothelial cells that had undergone a venous-to-arterial transition to rescue PAA formation, reducing CHD penetrance via a previously unknown intrinsic compensatory mechanism. In a study reporting digenic inheritance patterns, pathogenic variants of *NODAL* and *TBX20* were coinherited in siblings with CHD [21]. Both variants were predicted to be loss-of-function and compound mutant mice harboring mutations in both *Tbx20* and *Nodal* showed much more severe cardiac defects compared to littermate controls of other genotypes. This, like the work of Teekakirikul et al., support a digenic etiology for CHD, whereby a second genetic variant/perturbation is required for CHD manifestation as defective development may be partially rescued by innate developmental and compensatory mechanisms. Furthermore, the model of digenic inheritance was recently tested on a larger scale using exome sequencing data from 3,910 CHD parent-offspring trios and 3,644 control trios [22]. Statistical modeling and burden testing identified 10 gene pairs that were significantly enriched in the CHD cohort that may be implicated in digenic inheritance. This is especially interesting in the context of CHD risk alleles or CHD associated single nucleotide polymorphisms (SNP) identified in genome-wide association studies (GWAS) [23]. Similar work has been done in more specific circumstances such as in trabecular development in the context of cardiomyopathy [24]. Accordingly, GWAS will be an increasingly important resource in identifying disease associated genes/alleles and their coinheritance patterns. Together, this supports the idea that multiple disease associated alleles (i.e., multiple “hits”) may be required to produce a defect that cannot be rescued during development by compensatory mechanisms.

### Noncoding Variation

Utilization of genome sequencing has enabled identification of several potentially pathogenic variants in noncoding sequences that may contribute to disease [25]. Sophisticated machine learning algorithms are now able to pull from multiomics data, particularly related to chromatin architecture during mammalian cardiac development, to identify potentially pathogenic variation in regulatory element sequences (e.g. enhancers) [25, 26]. Functional assessment of these noncoding regions can be performed using massively parallel reporter assays using in vitro iPSC based systems as was done by Xiao et al. [27]. However, validation studies in vivo are challenging, likely due to limited cross-species conservation among non-coding regulatory sequences. As such, an atlas of highly conserved noncoding sequences is crucial in order to test potentially pathogenic variation in noncoding sequences [28, 29]. Recently, Yamaguchi et al. identified an *Nkx2-5* enhancer that is required for cardiac outflow tract development, and mice with homozygous loss of this enhancer have a full spectrum of CHD [30]. Moreover, a highly conserved intragenic enhancer found in titin was shown to be required for sarcomere formation in human iPSCs and a rare variant affecting this region was found to be associated with dilated cardiomyopathy, implying that regulatory elements and mutations to them are of novel clinical relevance [31]. In addition to regulatory element sequences, long noncoding RNA (lncRNA) are also known to regulate gene expression and mammalian cardiac development [32]. In fact, most CNVs implicated in CHD also affect lncRNAs present in those regions (dubbed CNV-lncRNAs). Lu et al. found that CNV-lncRNA in non-syndromic CHD are associated with CHD gene network hubs, suggesting that CNV-lncRNA may contribute to CHD etiologies [33]. Functional validation of one hub CNV-lncRNA, *HSALNG0104472*, was performed by RNAi mediated silencing in iPSCs, resulting in impaired differentiation and function of cardiomyocytes. Finally, noncoding regions also include untranslated intronic sequences that influence expression of specific isoforms required for cardiac development. A novel intronic variant in the transcription factor, ZIC3, results in a cryptic splice acceptor between exons encoding the 3’UTR, aberrantly affecting splicing and representing a non-traditional causal mechanism for monogenic CHD [34]. Moreover, specific miRNAs have been shown to be upregulated in the blood serum of mothers carrying a fetus with single ventricle disease, although the source of these miRNAs has not been determined and it remains unclear whether this is due to maternal or fetal genetic effects [35]. Additionally, upregulation of miR-187 expression was found in the hearts of fetuses with tetralogy of Fallot, and this was mechanistically found to impair cardiac endothelial development leading to CHD in mice suggesting that miRNAs also represent a form of noncoding sequence that may be susceptible to pathologic variation [36].

As we continue to generate large multiomic datasets informing cardiac development and disease, machine learning approaches are also expected to advance in parallel, aiding in the identification of novel genetic mechanisms underlying CHD. In fact, a recently published study used machine learning and single cell epigenomic data to prioritize noncoding variants in CHD [37]. Integration of such sophisticated machine learning technologies along with appropriate statistical modeling tools and omics data will improve the framework for CHD variant detection and greatly contribute to our understanding of genetic etiologies of CHD.

## Environmental Etiologies of CHD

Environmental factors contribute to approximately ~ 10% of CHD cases. Similarly to genetic factors, environmental contributors are heterogeneous and can be broadly classified into extrinsic and intrinsic factors (Fig. 2B).

### Extrinsic Factors

Several environmental exposures are known to have teratogenic effects on cardiac development, leading to increased incidence of CHD. Epidemiological studies across the globe have uncovered some of the most prominent environmental risk factors for CHD including prenatal exposure to heavy metals (lead, cadmium, mercury, arsenic), drugs (thalidomide, anti-convulsants, anti-depressants), nutritional deficiencies (folic acid/vitamin B12, Iron), retinol/Vitamin A, and alcohol consumption [8]. Historically, depending on the extent of these exposures within the population, public health motivated legislative measures are taken, and an informed lifestyle can modify the risk of exposure to such environmental factors and reduce the incidence of associated CHD. However, rapid urbanization, climate change, and increased reliance on technology are contributing to new public health concerns that may continue to contribute to the global incidence of CHD. As of late, air pollution is becoming among the most pervasive extrinsic environmental risk factor for disease. Several studies have identified an association between CHD and maternal exposure to fine particulate matter with diameter less than 2.5 μm (PM2.5) [38, 39]. PM2.5 has been shown to activate aryl-hydrocarbon receptors, leading to increased oxidative stress and mitochondrial dysfunction, and resulting in cardiac malformations in zebrafish [40–42]. Another major pollutant in modern times is micro- and nano-plastics, which may be manufactured as such or be a breakdown product of larger plastic material. Although there is no epidemiological data linking micro/nanoplastic exposure to CHD specifically, a recent investigation using chick embryos showed that nanoplastics are capable of dysregulating neural crest cell migration, resulting in congenital cardiac and craniofacial abnormalities [43]. The rapid transformation of society and its subsequent influence on our environment and lifestyle will continually give rise to other unknown CHD risk factors and as such, epidemiological studies must continue to identify and confirm new insults to develop prophylactic measures.

### Intrinsic Factors

Intrinsic factors refer to internal physiologic conditions during embryogenesis, including maternal age, illnesses and infections. These are generally considered less modifiable than the external environment and represent the complex interplay of biological processes that occur between the maternal environment and the fetus during development. The rising epidemic of diabetes mellitus, particularly in women of childbearing age, has made maternal diabetes among the most studied intrinsic risk factor for CHD [44, 45]. Studies using rodent models of maternal diabetes and single cell transcriptomic and epigenetic profiling has identified several embryonic cardiac developmental processes and cardiac cell lineages that are impaired under these conditions [46–49]. Although hyperglycemia is considered the primary manifestation, diabetes mellitus is a complex metabolic disorder that may affect cardiac development independent of glucose levels. In fact, impaired maternal metabolism via maternal microbiome profiling has been linked with several subtypes of CHD, indicative of an unfavorable maternal environment during cardiogenesis [50–52]. Similarly, maternal obesity has also been independently linked to CHD [53, 54]. Other maternal disease states include infections such as rubella and cytomegalovirus. Recently, an increased incidence of CHD was reported in cases of maternal COVID-19 infections [55]. Moreover, recent studies suggest that abnormal function of the placenta is sufficient to lead to CHD and there exists a placenta-heart axis that must be maintained for normal cardiac development. As an example of this, deletion of *Slc25a1 *in the trophoblast but not in the embryo, leads to CHD in mice [56]. This holds true for other genes, however, this model has not been proven in humans [57, 58]. Placental inflammation has also been implicated in CHD pathogenesis and placental abnormalities have been detected in pregnancies affected by CHD, although it is unclear whether this is a secondary effect of cardiac maldevelopment or serves a more causal role in CHD [59, 60]. Lastly, empirical evidence for gene-environment interactions, which have long been proposed to contribute to CHD, are finally being generated using animal models [61]. Studies have shown that haploinsufficiency of *Notch1* or *Nkx2-5* results in higher incidence of CHD with exposure to maternal diabetes or hypoxia [62–66]. However, clinical investigations in this regard have been limited, and investigation of a maternal diabetes associated CHD cohort did not identify any *de novo* pathogenic variation in the probands [67]. It is more likely that these interactions occur between rare recessive variants and environmental factors in a graded manner, consistent with the multiple-hit hypothesis described in the previous section.

## Treatment Options

Over the last several decades, multiple staged surgical interventions during early childhood have made even critical CHDs more survivable. However, as the heart is the first organ to form during embryogenesis, there is little opportunity for intervention to “fix” cardiac defects *in utero* and the best options to reduce CHD incidence at birth are primordial prevention strategies. In cases where a disease-causing variant is known, preimplantation screening and in vitro fertilization (IVF) are valuable tools for adult CHD patients who desire to have children unaffected by CHD [68]. There have also been recent advancements in utilizing gene and stem cell therapies to treat cardiovascular disease including genetically mediated cardiomyopathy. Gene therapies utilizing adeno-associated virus (AAVs) have been shown to ameliorate arrhythmogenic right ventricular cardiomyopathy in a patient-derived mouse knock-in model [69]. In patients and mice with an intronic splice site mutation in *PKP2*, arrhythmogenic right ventricular cardiomyopathy occurs through degradation of cell-cell junctions (i.e., desmosomes). In this knock-in model, postnatal and adult injection of AAVs carrying wildtype *Pkp2* rescued desmosome degradation and disease progression. Furthermore, genetic variants, such as *MYH6*, have been reported to be associated with poor clinical outcomes in patients with single ventricle heart disease [70]. Identification of such variants raise the possibility of postnatal gene therapy approaches to correct genetic deficiencies that have the potential to impact clinical outcomes and survival. In another study, therapeutic use of human umbilical cord mesenchymal stem cells from an unaffected sibling has been used to treat vessel loss from pulmonary arterial hypertension due to a genetic etiology, a *de novo ACVRL1* missense variant [71]. These studies demonstrate the potential application of gene therapy and/or stem cells to treat aspects of CHD and its associated complications.

In contrast, certain small molecule drugs and antioxidant strategies have been shown to work in mice in the context of maternal diabetes associated CHD. Teriflunomide, an FDA-approved drug, and echinacoside, a naturally occurring compound, can activate mitochondrial fusion and when injected in pregnant diabetic mice, reduce the incidence of CHD in mouse pups compared to non-diabetic controls [72]. Maternal diabetes associated oxidative stress is a major mechanism underlying impaired cardiac development and antioxidant supplementation during gestation has been shown to have varying degrees of success in reducing CHD in these cases. For instance, pharmacologic supplementation of an antioxidant, N-acetyl cysteine, has been shown to significantly reduce incidence of CHD in diabetic mice in one study but showed no discernable effects in another [66, 73]. Genetic strategies to mitigate cellular oxidative stress via overexpression *Sod1*, also show promising results in reducing CHD incidence in diabetic mice [74]. However, this strategy fails to work in presence of a gene-environment interaction between maternal diabetes and *Notch1* haploinsufficiency, suggesting genetic predisposition can attenuate response to therapeutic strategies [62]. Interestingly, maternal hyperoxia partially reduces the incidence of CHD in mice heterozygous for *Nkx2-5*, a gene known to interact with maternal hypoxia to increase the incidence of CHD [63, 65]. These studies further highlight the need to study gene-environment interactions underlying CHD as they will ultimately inform precision medicine-based approaches to reduce CHD risk in genetically sensitive populations.

## Conclusions

The last decade has been marked by remarkable advancement in our scientific understanding of CHD etiologies. From identifying novel monogenic, oligogenic, and noncoding disease-causing variants, to identifying molecular mechanisms underlying CHD associated with environment risk factors, we continue to reduce the percentage of unsolved CHD cases. As we bridge this gap in knowledge, future therapies for CHD may take the form of small molecules against druggable targets within dysregulated cardiac developmental pathways or gene therapies to replace nonfunctional etiologic genetic variants. Additionally, improved genetic screening and precision medicine approaches may enhance early diagnosis and personalized treatment strategies, ultimately improving outcomes for individuals affected by CHD.

## **Key References**

- Ramirez A, et al. Buffering mechanism in aortic arch artery formation and congenital heart disease. Circul Res. 2024;134(10):e112–32.
  - **It has long been proposed that during embryonic development, a buffering capacity exists to ensure robust development in the presence of environmental and genetic perturbation. Ramirez** ***et al*** **describe an innate compensatory mechanism that exists during murine aortic arch development to rescue abnormal pharyngeal arch artery morphogenesis and prevent the development of CHD.**
- Kars ME, et al. Deciphering the digenic architecture of congenital heart disease using trio exome sequencing data. Am J Hum Genet. 2025;112(3):583–98.
  - **Kars *et al *conducted trio exome sequencing of a large CHD cohort and identified deleterious variant pairs that are significantly associated with CHD. Novel analysis pipelines to decipher digenic and oligogenic variants associated with disease from genomic data will be essential in identifying new CHD etiologies.**
- Yuan X, et al. Maternal exposure to PM2. 5 and the risk of congenital heart defects in 1.4 million births: a nationwide surveillance-based study. Circulation. 2023;147(7):565–74.
  - **PM2.5 is considered a main pollutant that is released into the environment and inhaled. Across over a million births, Yuan** ***et al*** **determined that maternal exposure to these airborne particles is associated with an increased risk of CHD, especially when this exposure is during the months of preconception and continues to around eight weeks gestation.**

## Data Availability

No datasets were generated or analysed during the current study.
